# Case Report: High-grade supratentorial CNS neoplasm with *EP300::BCOR* fusion in an adult: expanding the morphologic and molecular spectrum of *BCOR*-fused tumors

**DOI:** 10.3389/fonc.2026.1809404

**Published:** 2026-09-03

**Authors:** Samer Abdulkader-Sande, Alberto Garrido, Andrea Durán-Cameselle, Concepción Fiaño-Valverde, Luis Ruiz-de Almiron, Brais Perez, Isaura Fernandez-Perez

**Affiliations:** 1 Department of Pathology, Alvaro Cunqueiro Hospital, Vigo, Spain; 2 Department of Medical Oncology, Alvaro Cunqueiro Hospital, Vigo, Spain; 3 Translational Oncology Research Group, Galicia Sur Health Research Institute (IISGS), Vigo, Spain

**Keywords:** adult, *BCOR* fusion, CNS tumor, *EP300::BCOR*, ependymoma-like, high-grade glioma-like, molecular profiling

## Abstract

Tumors of the central nervous system harboring alterations of *BCOR* comprise a heterogeneous and evolving group of neoplasms. CNS tumors with *BCOR* internal tandem duplication are recognized as a distinct WHO entity, whereas tumors harboring *EP300/CREBBP::BCOR/BCORL1* fusions represent an emerging and less clearly defined group with overlapping but non-identical morphologic and immunophenotypic features. We report a 42-year-old man with a supratentorial high-grade CNS neoplasm initially interpreted as a glial tumor with features suggestive of supratentorial anaplastic ependymoma. Histologically, the tumor showed marked intratumoral heterogeneity, with expansile/circumscribed ependymoma-like areas containing prominent perivascular pseudorosettes, and infiltrative high-grade areas with glioblastoma-like features, including extensive necrosis, marked microvascular proliferation, brisk mitotic activity and a Ki-67 index reaching approximately 70%. Immunohistochemistry showed GFAP negativity in the ependymoma-like areas and GFAP positivity in the high-grade glioblastoma-like component. OLIG2 was negative, EMA showed dot-like/paranuclear staining, and BCOR demonstrated moderate diffuse nuclear expression. RNA-based targeted sequencing identified reciprocal *EP300::BCOR* and *BCOR::EP300* fusion junctions. FISH demonstrated homozygous *CDKN2A/B* deletion and 1q32 gain, findings subsequently supported by DNA-based copy-number analysis within an aneuploid profile. No reportable SNV or indel alterations of established diagnostic significance were identified. DNA methylation profiling was not performed. Accordingly, the integrated diagnostic interpretation was based on morphology, immunophenotype and RNA-based fusion detection, without methylation-class confirmation. This case expands the recognized morphologic and immunophenotypic spectrum of CNS tumors with *EP300::BCOR* fusion and highlights the diagnostic value of fusion testing in ependymoma-like supratentorial tumors with additional high-grade glioma-like features.

## 1 Introduction

Tumors of the central nervous system (CNS) with alterations involving *BCOR* have emerged from the molecular reclassification of lesions historically grouped under poorly defined embryonal or primitive neuroectodermal categories (1, 2). In parallel, DNA methylation profiling has become an important tool for refining CNS tumor classification, particularly in diagnostically challenging neuroepithelial tumors (3). Among *BCOR*-related CNS tumors, CNS tumor with *BCOR* internal tandem duplication (*BCOR*-ITD) is recognized in the fifth edition of the WHO Classification of CNS Tumors as a distinct molecular entity within embryonal tumors (2). Classic *BCOR*-ITD tumors predominantly affect children and usually show a relatively circumscribed growth pattern, perivascular pseudorosettes, microcystic or myxoid change, necrosis, strong nuclear BCOR expression, absent or focal GFAP expression, and variable OLIG2 positivity (1, 4, 5).

More recently, CNS tumors harboring *BCOR* or *BCORL1* fusions, most often involving the chromatin-modifying genes *EP300* or *CREBBP*, have broadened the spectrum of *BCOR*-related CNS neoplasms (6–11). These tumors appear related to, but not identical with, classic *BCOR*-ITD tumors. Reported cases show a wider age distribution, frequent supratentorial location, and variable morphology. Some are diffusely infiltrative and glioma-like, whereas others are more circumscribed and display ependymoma-like architecture with perivascular pseudorosettes (6–8, 10). This diversity is also reflected immunohistochemically, with variable expression of GFAP, OLIG2, EMA and BCOR, limiting the reliability of any single marker as a diagnostic surrogate (8–10).

This overlap creates a challenging differential diagnosis, particularly with supratentorial ependymoma and adult-type diffuse high-grade glioma (6, 7, 10, 12). In such cases, integrated histological, immunohistochemical and molecular assessment is essential, especially when an ependymoma-like tumor lacks canonical ependymoma-associated alterations or displays additional high-grade glioma-like features.

We report a diagnostically challenging supratentorial high-grade CNS neoplasm in a 42-year-old man harboring a reciprocal *EP300::BCOR*/*BCOR::EP300* fusion. The tumor combined expansile ependymoma-like areas and infiltrative high-grade glioma-like regions with compartment-specific GFAP expression. This juxtaposition, together with the unusual fusion breakpoint configuration, illustrates the morphologic, immunophenotypic and molecular breadth of this emerging group.

## 2 Case description

**Figure 1:**
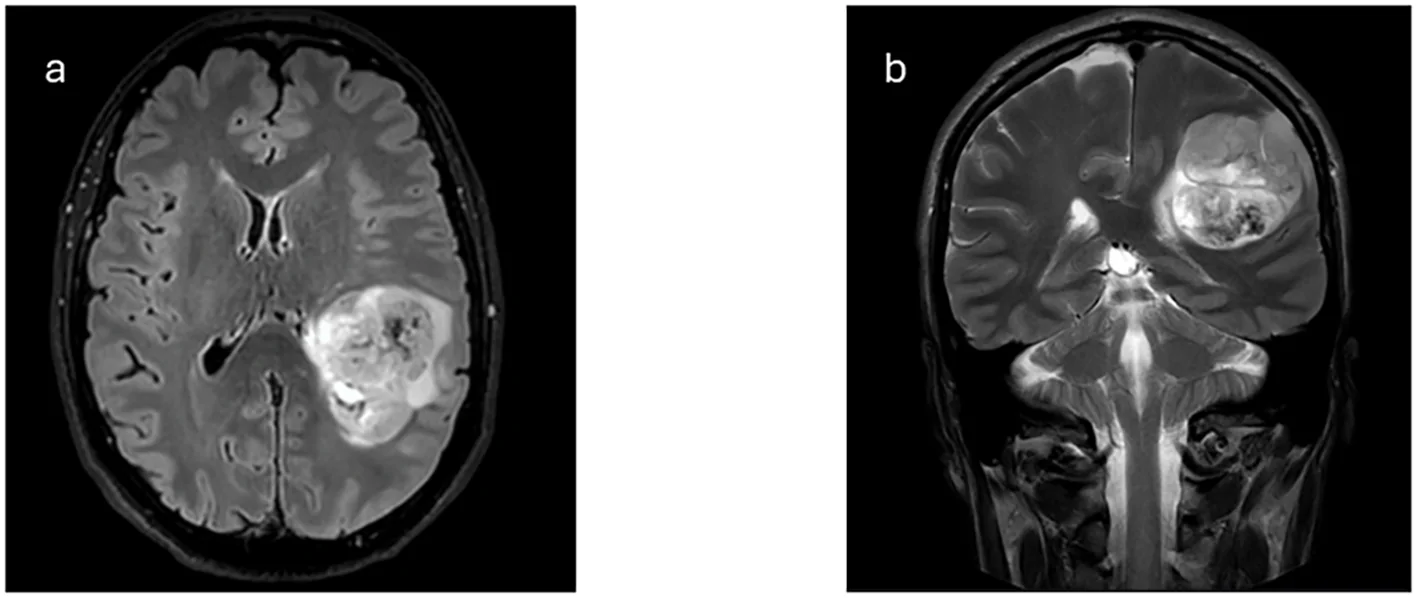
Preoperative brain MRI. **(a)** Axial FLAIR and **(b)** coronal T2 images showing a 60 × 45 × 45 mm left frontoparietal intra-axial mass with extensive cystic-necrotic components, surrounding vasogenic edema and associated mass effect.

A 42-year-old man with no relevant past medical history or known family history of cancer presented with progressive language impairment, memory difficulties, dysgraphia, reading difficulties and fine motor clumsiness. Four weeks after symptom onset, brain magnetic resonance imaging demonstrated a 60 × 45 × 45 mm left frontoparietal, predominantly parietal, intra-axial mass with probable hemorrhagic changes, extensive cystic-necrotic components, peripheral linear enhancement, solid nodular areas with diffusion restriction and increased cerebral blood volume, vasogenic edema and associated mass effect (**Figures 1A, B**). Radiologically, a high-grade glioma was suspected. Gross total surgical resection was performed approximately five weeks after symptom onset.

**Figure 2:**
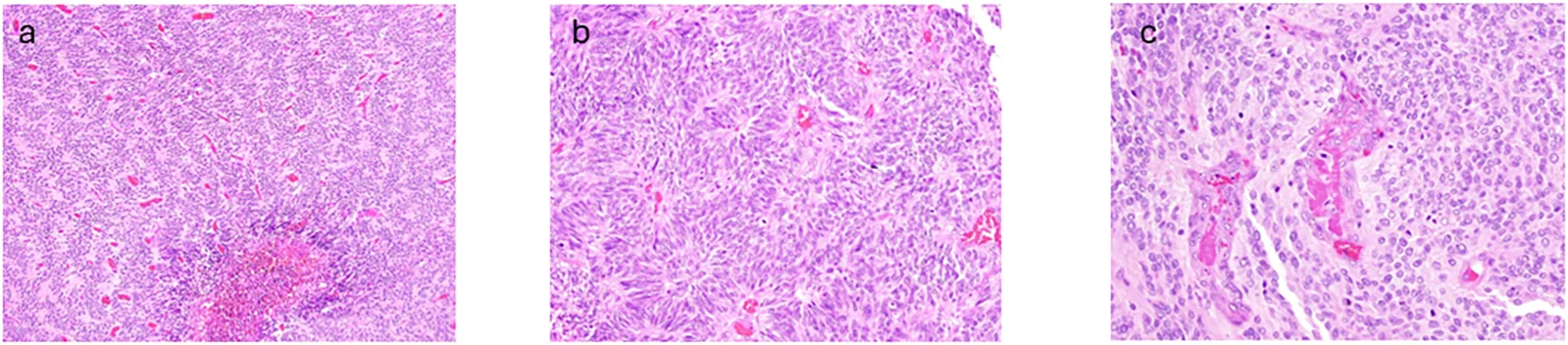
Histopathological features. **(a)** Ependymoma-like area with prominent perivascular pseudorosettes and adjacent necrosis (HE, 100x). **(b)** Densely cellular ribbon-like area composed of ovoid tumor cells with scant cytoplasm (HE, 100x). **(c)** Relatively monomorphic tumor cells with brisk mitotic activity and prominent microvascular proliferation (HE, 200x).

Initial histopathological assessment at the referring institution showed a high-grade glial/neuroepithelial neoplasm with features suggestive of supratentorial anaplastic ependymoma. The tumor was morphologically heterogeneous. Some areas showed a relatively expansile and partly circumscribed nodular growth pattern with prominent perivascular pseudorosettes, supporting an ependymoma-like interpretation (**Figure 2A**). True ependymal rosettes and ependymal canals were not identified. Other areas showed infiltrative and densely cellular growth and displayed frankly high-grade features, including extensive necrosis, marked microvascular proliferation, brisk mitotic activity and a high proliferative index (**Figures 2B, C**).

**Figure 3:**
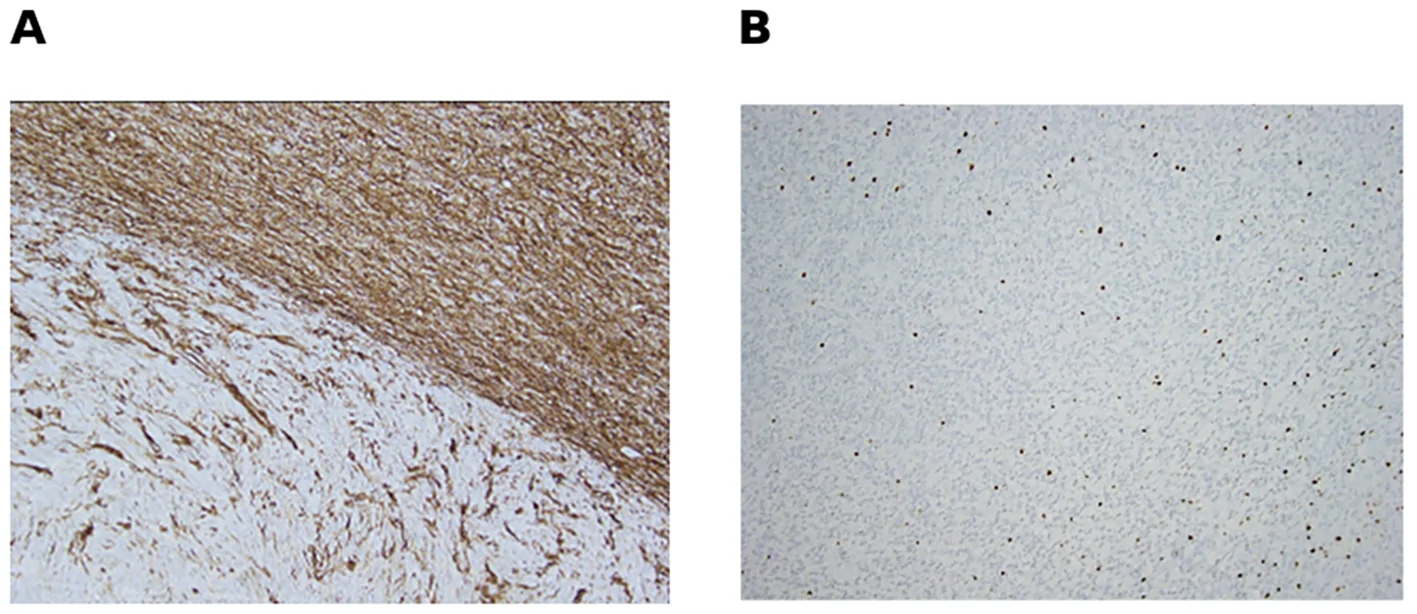
Compartment-specific GFAP expression and OLIG2 negativity. **(a)** Ependymoma-like area lacking GFAP expression and infiltrative high-grade glioma-like component showing strong and diffuse cytoplasmic GFAP staining (IHC, 100x). **(b)** Absence of OLIG2 expression in neoplastic cells (IHC, 100x).

The immunophenotype was also heterogeneous and paralleled the morphological variation. GFAP was negative in the ependymoma-like areas, particularly in the regions with perivascular pseudorosettes, whereas the infiltrative high-grade component showed GFAP expression and was interpreted as glioblastoma-like (**Figure 3A**). EMA showed dot-like/paranuclear staining, further supporting the initial consideration of ependymoma. OLIG2 was negative in the neoplastic cells (**Figure 3B**). Additional immunohistochemical findings included retained ATRX expression, negative IDH1 R132H immunostaining, retained H3K27me3 expression, negative H3K27M, and loss of p16 expression. L1CAM was negative. BCOR immunohistochemistry, performed during the subsequent diagnostic work-up, showed moderate diffuse nuclear expression.

Ancillary studies performed at the referring institution showed no *EGFR* amplification, *RELA*/C11orf95 rearrangement or 1p/19q codeletion. *BRAF* testing using an Idylla assay was negative. Given the combination of ependymoma-like architecture, infiltrative high-grade glioma-like areas and a non-canonical immunophenotype, the case was referred to a national reference center for ependymal tumors for expert review and additional molecular characterization.

RNA-based targeted next-generation sequencing using the FusionPlex Sarcoma v2 panel was performed at the national reference center. According to the validated molecular report, two reciprocal fusion junctions were identified and annotated as *EP300* exon 30::*BCOR* exon 11 and *BCOR* intron 10::*EP300* exon 31. No *YAP1* fusion was detected in the RNA fusion panel. Subsequent FISH studies demonstrated homozygous *CDKN2A/B* deletion and 1q32 gain. The homozygous deletion was concordant with the loss of p16 expression observed during the initial immunohistochemical work-up. DNA methylation profiling was not performed and no methylation class assignment was made.

On integrated review, the diagnosis was revised to a high-grade supratentorial CNS neoplasm harboring reciprocal *EP300::BCOR* and *BCOR::EP300* fusion transcripts, with ependymoma-like architecture and high-grade glioma-like features. Retrospective DNA-based NGS identified no reportable SNV or indel alterations of established diagnostic significance, including *IDH1/2* or *TERT* promoter mutations. Copy-number analysis showed an aneuploid profile with distal 1q gain, broad gains of chromosomes 2, 5 and 7, and a focal deep deletion of 9p21.3 encompassing *CDKN2A/CDKN2B*, consistent with the homozygous deletion demonstrated by FISH. EGFR amplification, whole-chromosome 10 loss and 1p/19q codeletion were absent.

Postoperatively, right upper limb paresis and coordination improved. Leptomeningeal involvement was ruled out by MRI and cerebrospinal fluid analysis before adjuvant focal radiotherapy. The patient received intensity-modulated radiotherapy without craniospinal irradiation, delivered in 28 fractions with a total dose of 61.6 Gy to the surgical bed and 50.45 Gy to the lower-dose volume. At month 8, local recurrence at the margins of the surgical bed was detected and considered unresectable. Carboplatin/etoposide chemotherapy was administered every 21 days, with carboplatin AUC 5 on day 1 and etoposide 100 mg/m² on days 1–3. Treatment was well tolerated, with only grade I thrombocytopenia. A partial radiological response was observed after three cycles, with maintained response and clinical stability after six cycles. Three months after completion of chemotherapy, clinical deterioration and radiological progression were documented. Bevacizumab was subsequently initiated; after three cycles, the patient showed further clinical progression with declining Karnofsky performance status and died 22 months after diagnosis. The main clinical, diagnostic and therapeutic events are summarized in **Table 1**.

**Table 1:** Timeline of clinical course, diagnostic work-up and treatment.

Time point	Event
Week 0	Onset of language impairment, memory difficulties, dysgraphia, reading difficulties and fine motor clumsiness.
Week 4	Brain MRI showed a 60 × 45 × 45 mm left frontoparietal intra-axial mass with cystic-necrotic components and mass effect.
Week 5	Gross total surgical resection of the lesion was performed.
Week 6	Initial histopathological assessment favored a high-grade glial neoplasm with features compatible with supratentorial anaplastic ependymoma.
Week 8	Integrated molecular work-up identified a reciprocal *EP300::BCOR*/*BCOR::EP300* fusion by RNA-based targeted sequencing. FISH showed homozygous *CDKN2A/B* deletion and 1q32 gain. DNA methylation profiling was not performed.
Month 3-4	Intensity-modulated focal radiotherapy was delivered in 28 fractions, with 61.6 Gy to the surgical bed and 50.45 Gy to the lower-dose volume, without craniospinal irradiation.
Month 8	Follow-up MRI showed unresectable local recurrence at the margins of the surgical bed.
Month 9-14	Carboplatin AUC 5 day 1 + etoposide 100 mg/m² days 1–3 q21 days was administered, achieving partial radiological response after 3 cycles and clinical stability after 6 cycles.
Month 17	Radiological and clinical disease progression was observed.
Month 18-21	Bevacizumab was initiated, followed by progression after 3 cycles with clinical deterioration.
Month 22	The patient died 22 months after diagnosis.
Retrospective molecular work-up	DNA-based NGS identified no reportable SNV or indel alterations of established diagnostic significance, including *IDH1/2* or *TERT* promoter mutations. Copy-number analysis showed an aneuploid profile with distal 1q gain, broad gains of chromosomes 2, 5 and 7, and a focal deep 9p21.3 deletion encompassing *CDKN2A/CDKN2B*, consistent with homozygous deletion and concordant with the previous FISH findings. *EGFR* amplification, whole-chromosome 10 loss and 1p/19q codeletion were absent.

## 3 Discussion

The present case illustrates a diagnostic scenario in which an *EP300::BCOR*-fused CNS neoplasm closely mimicked a supratentorial ependymoma at initial assessment. The tumor was supratentorial and contained relatively expansile/circumscribed areas with prominent perivascular pseudorosettes. In these regions, EMA showed a dot-like/paranuclear pattern and GFAP expression was absent, further supporting an ependymoma-like phenotype. However, true ependymal rosettes and ependymal canals were not identified. Moreover, FISH for *RELA*/C11orf95 rearrangement, L1CAM immunohistochemistry and RNA-based testing for *YAP1* fusion were negative. Broader molecular characterization was therefore essential and identified reciprocal *EP300::BCOR* and *BCOR::EP300* fusion transcripts. Similar diagnostic pitfalls have been described in *BCOR/BCORL1*-fused CNS tumors, which may initially be interpreted as ependymoma, glioma, glioneuronal tumor or another high-grade neuroepithelial neoplasm (6–8, 10, 12). Circumscribed growth, perivascular pseudorosettes and microcystic or myxoid change appear particularly likely to suggest an ependymal phenotype (7, 8, 10, 13).

A major feature of the present tumor was the coexistence of a second, infiltrative high-grade glioma-like component. These areas showed extensive necrosis, marked microvascular proliferation, brisk mitotic activity and a Ki-67 index reaching approximately 70%. GFAP expression was restricted to this infiltrative component, whereas the ependymoma-like areas remained negative, establishing a close spatial relationship between morphology and immunophenotype. Retrospective DNA-based NGS did not identify *IDH1/2* or *TERT* promoter mutations or other reportable SNV/indel alterations of established diagnostic significance. Copy-number analysis showed chromosome 7 gain without accompanying whole-chromosome 10 loss and *EGFR* amplification was absent. In this context, the molecular findings did not support assignment to a conventional adult-type diffuse glioma category. Instead, the reciprocal *EP300::BCOR*/*BCOR::EP300* fusion remained the principal molecular alteration supporting the descriptive integrated diagnosis.

The literature on CNS tumors with *EP300/CREBBP::BCOR/BCORL1* fusions provides a useful, although still incomplete, framework for interpreting this case. The earliest *EP300::BCOR* gliomas reported by Torre et al. showed a predominantly infiltrative glioma-like pattern, with GFAP and OLIG2 expression and BCOR nuclear immunoreactivity, but lacked prominent perivascular pseudorosettes (6). In contrast, the tumors reported by Tauziède-Espariat et al. showed a more circumscribed, HGNET-*BCOR*-like or ependymoma-like morphology, with pseudorosettes, microcysts, high-grade features, GFAP negativity and diffuse OLIG2 expression. These tumors clustered with HGNET-*BCOR* by methylation profiling and harbored *EP300::BCOR* fusions involving *EP300* exon 31 and *BCOR* exon 4 (7). Sugino et al. subsequently described an adult tumor with an anaplastic ependymoma-like morphology and *EP300::BCOR* fusion, further illustrating the potential for this group to mimic ependymoma (10). Wu et al. broadened the clinicopathologic spectrum by showing that *EP300::BCOR*-fused tumors may occur across a wide age range, are often supratentorial and may display either infiltrative glioma-like or more circumscribed ependymoma-like growth patterns (8).

Related alterations involving *CREBBP*, *BCOR* and *BCORL1* further illustrate the heterogeneity of this emerging group. Yamazaki et al. described a diffusely infiltrating glioma with *CREBBP::BCORL1* fusion, diffuse OLIG2 expression, scattered GFAP positivity and diffuse BCOR immunoreactivity (9). Subsequent reports, including those of Barresi et al. and Ebrahimi et al., added examples with *CREBBP::BCORL1* or *BCOR::CREBBP* fusions and variable ependymoma-like or malignant neuroepithelial morphology (11, 14). More recently, Lou et al. reported a CNS tumor assigned to the BCOR/BCORL1-fusion methylation class despite the absence of a demonstrated fusion, further illustrating that the molecular boundaries of this emerging group remain incompletely defined (15). Overall, the present tumor overlaps with previously reported cases but does not fit exclusively within either the infiltrative glioma-like or the circumscribed ependymoma-like pattern. Instead, both components were represented within the same neoplasm.

The immunophenotypic findings also support marked intratumoral heterogeneity. The ependymoma-like areas, characterized by expansile growth and perivascular pseudorosettes, were GFAP-negative and showed dot-like/paranuclear EMA staining. Conversely, the infiltrative high-grade glioma-like areas expressed GFAP in keeping with their glial morphology. Published tumors have similarly shown variable GFAP expression, ranging from absent or focal staining in more circumscribed examples to more conspicuous expression in infiltrative glioma-like lesions. EMA expression is also variable and should not be considered a defining feature. OLIG2 negativity was noteworthy, because several previously reported *EP300::BCOR*- and *CREBBP/BCORL1*-related tumors showed focal or diffuse OLIG2 expression.

BCOR immunohistochemistry should likewise be interpreted cautiously. Moderate diffuse nuclear expression was present in this case, but BCOR staining is neither uniformly expressed nor specific for a particular *BCOR*-related genetic alteration. Negative BCOR immunoreactivity in some *EP300::BCOR*-fused tumors has been proposed to reflect loss of the N-terminal epitope recognized by commonly used antibodies, depending on the fusion breakpoint. Conversely, BCOR positivity has also been documented in *BCORL1*-fused tumors without *BCOR* rearrangement or internal tandem duplication, potentially reflecting altered expression or regulatory effects (6–11, 14). BCOR immunohistochemistry should therefore be regarded as a supportive finding rather than as a surrogate for fusion, rearrangement or *BCOR*-ITD.

The breakpoint configuration reported in the present tumor also differed from the more frequently described *EP300::BCOR* junctions in this tumor group (7, 8, 10). Although its functional significance remains unknown, documentation of this distinct junction pattern broadens the limited molecular spectrum reported for *EP300::BCOR*-fused CNS tumors.

The main limitation of this report is the absence of DNA methylation profiling, which prevented assignment to a specific epigenetic class. Accordingly, the integrated diagnosis in the present case is descriptive and rests on the combined morphologic and immunophenotypic findings together with RNA-based detection of the *EP300::BCOR*/*BCOR::EP300* fusion, without methylation-class confirmation. Retrospective DNA-based NGS nevertheless provided additional molecular characterization, demonstrating an aneuploid copy-number profile with distal 1q gain, broad gains of chromosomes 2, 5 and 7, and focal homozygous deletion of *CDKN2A/B*. No *IDH1/2* or *TERT* promoter mutations, *EGFR* amplification or whole-chromosome 10 loss were identified. Histone *H3* genes were not included in the DNA panel, and *H3* G34 status was therefore not directly assessed by sequencing. The diagnostic and prognostic significance of *CDKN2A/B* homozygous deletion and distal 1q gain in *EP300::BCOR*-fused tumors remains to be established.

Therapeutic evidence for CNS tumors with *BCOR/BCORL1* fusions remains limited and management has generally been guided by histologic interpretation and individual clinical circumstances rather than by prospective evidence (8, 13). In the present patient, carboplatin/etoposide produced a partial radiological response and temporary clinical stability, followed by subsequent progression, whereas bevacizumab did not achieve sustained disease control. Published experience in *BCOR*-ITD tumors provides additional clinical context, although these tumors should not be considered therapeutically equivalent to *EP300::BCOR*-fused neoplasms (16).

Taken together, this case expands the morphologic, immunophenotypic and molecular spectrum of *EP300::BCOR*-fused CNS tumors by documenting spatially distinct ependymoma-like and infiltrative high-grade glioma-like components within the same neoplasm. From a practical diagnostic perspective, RNA-based fusion testing should be considered in supratentorial ependymoma-like tumors with a discordant immunophenotype, absent canonical ependymoma-associated alterations or an additional infiltrative high-grade glioma-like component.

## 4 Patient perspective

The patient perspective could not be obtained because the patient died before preparation of this report. All clinical and pathological information was fully de-identified, and consent/ethical procedures were followed according to institutional and journal requirements.

## Data Availability

The original contributions presented in the study are included in the article/supplementary material. Further inquiries can be directed to the corresponding author.
